# Mindful eating under pressure in combat sport: a single-case study of an adolescent athlete

**DOI:** 10.3389/fpsyg.2025.1624709

**Published:** 2025-10-27

**Authors:** Merve Rumeysa Alpay, Zekihan Hazar, Kürşat Hazar, Reka Erika Kovacs, Szilvia Boros

**Affiliations:** ^1^Institute of Health Sciences, Faculty of Sport Science, Sivas Cumhuriyet University, Sivas, Türkiye; ^2^Physical Education and Sports Teaching, Faculty of Sports Sciences, Ağrı Ibrahim Çeçen University, Ağrı, Türkiye; ^3^Faculty of Health and Sport Sciences, Psychology and Health Management, Széchenyi University, Györ, Hungary

**Keywords:** mindfulness intervention, combat sports, mindful eating, weight control, under pressure, single-case study

## Abstract

**Objectives:**

The purpose of this study was to examine closely how mindful eating intervention influences the eating behavior of a kickboxer 10 days before the competition.

**Methods:**

A mindful intervention was conducted. A mixed method was used, in which data was collected from two semi-structured interviews and four scales [Eating Attitudes Test (EAT-26), Eating Disorder Examination Questionnaire (EDE-Q), Dutch Eating Behavior Questionnaire (DEBQ), and Mindful Eating Scale (MEQ-30)]. The first semi-structured interview and four scales were administered before the intervention process, and the last interview was conducted after the competition. 10 days before the competition, 10 sessions of “mindful raisin eating” exercise, each lasting 10 min, were performed. The scores obtained from the scales were calculated manually. The data collected from semi-structured interviews were analyzed using the descriptive analysis method.

**Results:**

According to the administered 4 scales initially, the athlete’s average scores were (X̄=16) on the Eating Attitude Test (EAT-26), (X̄=3.125) on the “shape concern” sub-dimension of the Eating Disorder Examination Questionnaire (EDE-Q), (X̄=4) on the Emotional Eating sub-dimension of the Dutch Eating Behavior Questionnaire (DEBQ), and (X̄=2.5) on both the “awareness” and “eating control” sub-dimensions of the Mindful Eating Questionnaire (MEQ-30). Qualitative data showed, positive improvements were detected in eating attitudes and behaviors, stress management, perceived performance, and body image, respectively. During the mindful eating exercises, she lost approximately 2.4 kg (~3.9% of her body weight) without experiencing stress on weigh-in day. She also reported that focusing on mindful eating helped her avoid unhealthy foods and made her feel safe and calm.

**Conclusion:**

It was stated by the athlete that there were positive improvements in eating attitudes and behaviors, level of coping with stress, perceived performance and body perception.

## Introduction

Weight control is a critical issue in combat sports, where athletes are often required to compete in weight classes lower than their natural body weight. This leads many athletes, particularly elite-level competitors, to engage in rapid weight loss strategies shortly before competitions (Kim and Park, 2020; Ranisavljev et al., 2022; Pettersson et al., 2012). Common practices include dehydration, reduced food and fluid intake, increased training intensity, the use of saunas, and wearing plastic clothing to induce sweating (Lakicevic et al., 2021; Matthews et al., 2019; Khodaee et al., 2015). Although such methods may offer a short-term competitive advantage, they pose serious risks to both physical and mental health. Therefore, moderate and gradual weight loss—typically around 3 to 5% of body weight—is recommended to minimize performance deficits and health complications (Martínez-Aranda et al., 2023).

In recent years, mindful eating has gained attention in the field of sport psychology as a healthier alternative to traditional weight-cutting methods. Mindful eating involves eating slowly, using all five senses, and paying attention to the body’s hunger and fullness cues without judgment (Monroe, 2015; Lofgren, 2015; Miller, 2017). Rooted in the principles of mindfulness and Zen Buddhism, this approach emphasizes awareness and acceptance—of food, bodily sensations, and emotional experiences—during meals (Albers, 2010; Lyzwinski et al., 2019; Nelson, 2017). It helps individuals move away from automatic, emotionally-driven eating patterns and promotes a balanced relationship with food. Studies have shown that mindful eating reduces disordered eating behaviors such as binge eating, emotional eating, and restrictive eating, while also contributing to sustainable weight management (Godfrey et al., 2015; Mason et al., 2016; Dalen et al., 2010; Miller et al., 2012; Kristeller and Jordan, 2018; İnözü and Köse, 2023; McIver et al., 2009). This evidence is also consistent with studies using mindfulness-based practices to transform eating behaviors in adolescent populations. The systematic and meta-analytical studies reviewed have found improvements in eating behaviors in adolescents with mindfulness, a reduction in eating disorders, and an increase in emotional awareness (Omiwole et al., 2019).

Adolescent athletes, in particular, face unique challenges in maintaining healthy eating behaviors due to high training demands, body image concerns, and social pressure (Patel et al., 2003) These factors can increase the risk of impulsive and emotionally driven eating habits, especially under the psychological stress of competition (Giel et al., 2016; Mancine et al., 2020). Research supports that mindful eating can be beneficial in this population by fostering emotional regulation, self-awareness, and healthier food choices (Barnes and Kristeller, 2016; Hendrickson and Rasmussen, 2017). Moreover, mindful eating has been associated with improved athletic performance by supporting consistent energy levels and reducing the mental burden of food-related guilt and anxiety (Arthur-Cameselle, 2016; Pintado-Cucarella and Rodríguez-Salgado, 2016).

This case study aimed to explore the eating behaviors of an adolescent national-level combat sport athlete during the 10 days leading up to a major competition. The primary goals were to support stable weight control, increase adherence to a prescribed diet plan, reduce emotional distress related to eating, and enhance overall well-being. Through the implementation of mindful eating techniques, the athlete was encouraged to slow down her eating process, become aware of emotional triggers, and respond to hunger and fullness cues more accurately. Observing the athlete during this critical period provided insights into how mindfulness-based approaches can assist athletes in maintaining a healthy relationship with food under pressure, without compromising their physical performance or psychological health. In this study, the raisin exercise, which is regarded as a classical and frequently applied practice in mindful eating interventions, was utilized. It was selected due to its accessibility, appropriateness for introductory levels, and its capacity to engage multiple senses (Çolak and Aktaç, 2019). To the best of our knowledge, while raisin mindfulness exercises have been incorporated into certain sessions of mindfulness programs designed for performance athletes, no study has employed the same exercise consistently across all sessions as a standalone practice (Demarzo et al., 2015; De Petrillo et al., 2009).

## Sample, methods and design

### Athlete characteristics

This case study focuses on a 16-year-old female kickboxer who has been competing nationally and internationally for 5 years. She has achieved significant success, including two national championships, two third-place finishes, and one fifth-place finish at the European level. The study was designed to explore the psychological and behavioral challenges she faced in managing her weight before a competition, particularly the stress and pressure during the 20-day weight-cutting period before competing in the 60 kg category (initial weight: 61 kg, height: 165 cm). She did not employ any additional weight-cutting strategies (e.g., fluid restriction, training in sweat suits) during the study. Importantly, the athlete had not previously followed any structured dietary program under the supervision of a nutritionist.

After reporting high stress and familial pressure related to weight loss, a sports psychologist with expertise in mindfulness-based interventions conducted a mixed-method study. Ethical approval was granted by the Osmaniye Korkut Ata University Ethics Committee (E-58565088-100-191825), and informed consent was obtained.

An explanatory sequential design was used, combining quantitative and qualitative data. Four psychological scales—EAT-26, EDE-Q, DEBQ, and MEQ-30—were administered pre-intervention to assess clinical risk related to disordered eating and mindful eating habits. The EAT-26 measured general eating attitudes; the EDE-Q focused on recent clinical symptoms; the DEBQ evaluated emotional and external triggers for eating; and the MEQ-30 assessed mindfulness in eating behavior.

The intervention included semi-structured interviews (pre and post-competition) and mindfulness-based eating practices, specifically the “raisin exercise,” to enhance awareness and reduce impulsive eating. Drawing on previous research showing the benefits of mindfulness on adolescent eating behavior (e.g., Atkinson and Wade, 2015; Godfrey et al., 2015), the study aimed to observe whether mindfulness techniques could support healthier eating behavior under competitive pressure and help the athlete maintain stable weight.

### Intervention

#### Mindful raisin eating treatment summary

Raisin exercise is a practice that consciously brings attention to the act of eating by actively using the participant’s five senses. Participant focus on the color, smell, texture, taste and even sound of the raisin. This exercise asks the participant to approach the raisin with open curiosity and an open-minded way as if tasting it for the first time. Meanwhile, the participant is asked to accept the thoughts and feelings that come to his/her mind without judgment and to focus his/her attention on the raisin again (Kabat-Zinn, 2005).

The intervention was carried out for 10 days in a quiet school classroom, with each session lasting 10 min and only the participant and the researcher being present. In each session, the “raisin meditation” created by Jon Kabat-Zinn within the scope of the MBSR (Mindfulness-Based Stress Reduction) program was applied. This meditation is a mindful raisin eating exercise, a common mindful eating induction used in clinical and non-clinical settings (Kabat-Zinn, 2006). The participant listened to the Turkish video of this exercise (Doctor_uyku, 2021).

After the every session, the participant was given mindful eating homework sheets prepared by MIT Medical and was asked to fill them in at every meal. Homework papers were tracked with an attendance list. The homework sheets included the times when the athlete ate each meal, the foods she ate, a box numbered from 1 to 10 where the athlete could indicate her hunger level, and spaces where she could write what she felt and thought while eating (MIT Medical, 2024).

The expectation from the intervention was to ensure that the athlete could control her weight without experiencing weight stress until the match and to protect her from more unhealthy weight control behaviors (such as starving herself). It was also to relieve the feeling of guilt she felt after every meal.

**Figure 1:**
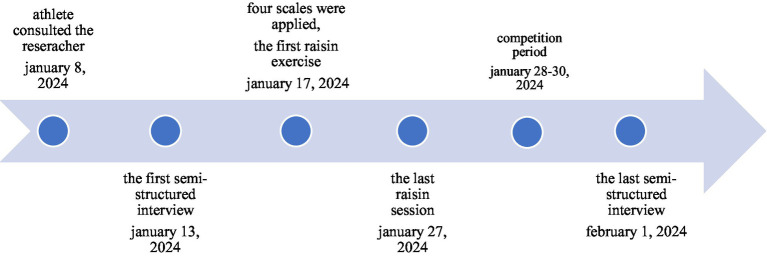
Timeline diagram.

The four scales administered before the intervention provided a comprehensive baseline assessment, but the main focus was to capture details in behavior and perception that cannot be reflected quantitatively in the athlete’s experience. Details of the intervention are presented in the timeline diagram (Figure 1).

#### Procedure: data collection

First, the participant was informed about the study and the use of the data, and then a consent form was obtained. The first semi-structured interview was conducted 5 days after the athlete consulted the researcher. A day of this semi-structured interview, four scales were applied to clarify whether the athlete had a clinical case (Eating Attitudes Test, EAT-26; Garner et al., 1982; Ergüney-Okumuş and Sertel-Berk, 2020), Eating Disorder Examination Questionnaire (EDE-Q; Fairburn and Beglin, 1994; Yucel et al., 2011), Dutch Eating Behavior Questionnaire (DEBQ; Van Strien et al., 1986; Bozan et al., 2011) and Mindful Eating Scale (MEQ-30; Baer et al., 2006; Köse et al., 2016). Three days after the application of the scales and their manual analysis and interpretation, a short introduction about mindfulness was given to the athlete before the first raisin exercise sessions. Information was given about what mindfulness is, in which areas it is used, attitudes, what mindful eating is and what will be done in these sessions. Then, the raisin exercise (Doctor_uyku, 2021) which is a 10-min mindful eating exercise, was applied every day (for 10 days) until the competition day. The participant listened to the Turkish video of this exercise (male voices). This intervention was carried out daily in a quiet room with the researcher. After each session, the athlete was given a mindful eating diary assignment sheet prepared by MIT Medical (MIT Medical, 2024).

On this homework sheet, the athlete was asked to practice the mindful eating attitudes she learned at home and reflect them on the sheet. She was asked to score from 1 to 10 how hungry she felt while eating and to note what she thought and felt. After a total of 10 raisin sessions, the athlete competed in the National Turkish Kickboxing competition. On the match day, no intervention was applied to the athlete, nor for the following 3 days (the competition lasted 3 days). At the end of the 10 sessions, 10 mindful eating journal worksheets were obtained. At the end of the competition, the second semi-structured interview was conducted.

The first semi-structured interview date with the participant was January 13, 2024. The first mindful raisin eating session was held on January 17, 2024. After 10 sessions, the sessions ended on January 27, 2024. The athlete participated in the match on January 28, 2024. The Türkiye kickboxing championship lasted 3 days. The last semi-structured interview date was held on February 1, 2024.

### Data analyzing

Since the number of participants was a person, the scores obtained from the scales were calculated manually for the analysis of quantitative data. The data collected from both semi-structured interviews were analyzed using the descriptive analysis method, one of the qualitative research methods. The descriptive analysis method, which is a type of qualitative data analysis, is the process of summarizing and interpreting data according to predetermined themes in order to understand the data and reveal their basic characteristics (Yıldırım and Şimşek, 2018, p. 239). Before the interviews, question themes were created by one dietitian, one medical doctor and one psychological counselor. After two semi-structured interviews, the audio recordings of the interviews were transcribed. Then, after 10 sessions, the reflective sentences taken from the athlete’s second interview transcripts were coded following predetermined themes. The interviews were coded by a single researcher. To enhance trustworthiness, codes and themes were reviewed and discussed with the dietitian, medical doctor, and psychological counselor who contributed to creating the question themes. The codes obtained from the semi-structured interviews were obtained only from the second interview. The first interview was conducted to obtain detailed information about the athlete’s eating attitudes and behaviors before the intervention. However, the main codes in the study were created only from the second interview to examine the experiences after the application.

## Results

### Quantitative results

According to the administered 4 scales initially, it was determined that the athlete did not have awareness and eating control behaviors because her scores from the “awareness” (X̄=2.5) and “eating control” (X̄= 2.5) subscales of the Mindful Eating Questionnaire (MEQ-30) were low. No abnormal eating behavior was detected as a result of the scores she received from the Eating Attitude Test (EAT-26; X̄=16). When the scores obtained from the Dutch Eating Behavior Questionnaire (DEBQ) were evaluated, it was seen that the athlete received high scores from the Emotional Eating subscale (X̄=4). Moreover, the athlete received a high score from the “shape concern” sub-dimension subscale of the Eating Disorder Examination Questionnaire (EDE-Q; X̄=3.125). Among these items, the athlete marked the option “every day” for the following items: eating secretly, feeling guilty after eating, and being seen by someone while eating, in the first semi-structured interview, she always emphasized that she felt guilty after eating.

After the mindful raisin exercises, the athlete reduced her weight from 61 to 58.6 in 10 days. She lost approximately 2.4 kg (~3.9% of her body weight). In order to compete, it was required for her to maintain a weight of 60 kilograms or less. The quantitative results of the study are shown in Table 1.

**Table 1:** Quantitative results.

Mindful eating questionnaire (MEQ-30)	Mean score (X̄)	Reference value / cut-off
Awareness	2.5	3 (Köse et al., 2016).
Eating control	2.5	3 (Köse et al., 2016).
Eating Attitude Survey (EAT-26)	16	20 (Ergüney-Okumuş and Sertel-Berk, 2020)
Eating Disorder Examination Questionnaire (EDE-Q)		
Shape concern (SC)	3.125	4 (Luce et al., 2008)
Dutch Eating Behavior Questionnaire (DEBQ)		
Emotional Eating	4	2.2 (Çamurcu, 2017).

### Qualitative results

After the first semi-structured interview, the athlete stated that she deprived herself of food even though she was hungry when she was approaching her all competition periods, that she was constantly overweight, and that her family forced her to starve. At the end of the first interview, it was concluded that she exhibited secret eating behaviors and was trained intensively because she felt guilty after eating. She also stated that she could not eat anything for the last 3 days before the competition and that she constantly wanted to vomit when she ate. The qualitative results of the study are shown in Table 2.

**Table 2:** Second interview responses.

Theme	Code	Quotation	Baseline quantitative context
Eating attitudes	Healthy Diet	“Mindful Eating… makes us feel we should avoid junk food, sugar, chips, and cola, and focus on healthy things.”	MEQ-30 Awareness subscale: M = 2.5 (Reference = 3) – low pre-test mindful eating attitudes. Athlete emphasized increased orientation toward mindful eating after training.
	Emotional Eating	“While other athletes had difficulty with weigh-in, I passed easily. The mindful raisin exercise decreased my hunger, and I lost weight more easily.”	DEBQ Emotional Eating subscale: M = 4 (Reference = 2.2) – high pre-test emotional eating. After training, athlete reported reduced hunger and easier weight control.
Stress factors	Stress Reduction Strategies	“During mindful eating exercises, I focused on the present and felt less stressed about the competition. My mood improved and I felt comfortable.”	MEQ-30 Eating Control subscale: M = 2.5 (Reference = 3) – low pre-test eating control. Mindful eating helped focus, reduce stress, and regulate eating behavior.
Performance	Perceived Sports Performance	“Eating healthy helped me maintain and even improve performance. Avoiding sugar and cola reduced fatigue.”	MEQ-30 Eating Control subscale: M = 2.5 (Reference = 3) – low pre-test eating control. Mindful eating supported healthy choices and performance.
Body perception	Appearance Anxiety	“I have some dissatisfaction, but I’m better than before. At least I’m not too pot-bellied.”	EDE-Q Shape Concern subscale: M = 3.125 (Reference = 4) – moderate pre-test shape concern. Athlete reported improvement in body shape perception.

Since the athlete did not exercise on the match day, the theme of ‘exercise routine close to the match’ was not addressed in the second interview question. The purpose of including this theme in the first interview questions was to learn the level of exercise intensity of the athlete close to the competition during the weight loss process.

An attempt was made to find out whether she had changed her exercise routine in an unhealthy way in to lose weight. This theme was replaced by the ‘perceived performance theme’ in the second semi-structured interview.

## Discussion

Overall, in this study, 10 sessions of “mindful raisin eating” exercise program, each lasting 10 min, were applied to minimize the difficulties experienced by the athlete in weight control under pressure. After the mindful raisin exercises, the athlete reduced her weight from 61 to 58.6 in 10 days. She pointed out after raisin exercise that she acquired conscious eating behavior, felt less hunger and lost weight more easily. She also remarked that her conscious eating behavior helped her stay away from junk food, like sugar, chips and cola and directed her to healthy foods. She defined that this situation improved her performance. While the athlete generally experienced an easier weight loss process with the eating behaviors and attitudes she acquired, she specifically managed the weight control pressure she experienced better and experienced a significant decrease in stress level. The athlete’s statements in the interview records confirm this situation.

It seems important to study the psychological dimensions of eating behaviors under pressure in athletes, because theyare suspicious about what and how much they should eat close to the competition. This mindfulness eating intervention was a short period, and there were limited studies in the literature where such short-term mindfulness interventions have been studied on a case-study basis on adolescent athletes. However, brief (five-minute) mindfulness-based interventions have been ascertained to lead to numerous health-related improvements (Howarth et al., 2019). In addition, studies have shown that besides mindfulness programs lasting 8 weeks with each session lasting 90 min, even one session of 10-min mindfulness practice applied for a short period improves instant performance under pressure (Wolch et al., 2021). There was evidence that mindfulness therapies create insight, even in a very short time (Howarth et al., 2019).

It has been proven that awareness-based eating studies affect individuals’ eating attitudes and behaviors (O'Reilly et al., 2014). In this regard, the athlete stated that mindful eating led him to make healthier eating choices by avoiding junk food. She stated that these exercises reduced his hunger, which helped her lose weight and made it easier for her to pass the weigh-in check before the match. Similar to the results of the study, Jordan et al. (2014) found in their study that mindfulness encourages a healthy diet by affecting impulsive eating behaviors and junk food consumption. Individuals who received mindfulness training preferred the less caloric foods offered to them.

Moreover, mindful eating is effective in reducing individuals’ stress levels, as it has a negative relationship with emotional eating (Pidgeon et al., 2013). As a matter of fact, in this study, she stated that she did the mindful raisin exercises even on match days and this helped her achieve a state of calm by temporarily changing her focus. She clarified that these exercises made her think less about the match, which reduced her anxiety and stress levels. She emphasized that her well-being was better than before. In parallel with the research results, in a randomized study conducted by Miller et al. (2021) on 29 adolescents at risk of obesity, it was concluded that mindfulness stimulation reduced the state anxiety response in adolescents, compared to neutral stimulation after 10 min of mindfulness and neutral induction.

It has been observed that mindfulness exercises effectively reduce overeating as well as emotional eating. At the end of the second semi-structured interview, the athlete stated that she felt less hungry after mindful eating exercises. Lastly, although she stated that she was under pressure from her mother about weight control, eating behavior and body appearance in the first session before the intervention, she did not make any comments about this at the end of the competition. This change in focus might suggest the potential impact of mindfulness practices on reducing concerns related to body dissatisfaction and eating behaviors. Studies have proven that mindfulness has a negative relationship with body dissatisfaction and eating disorder psychopathology (Sala et al., 2020). As a matter of fact, in this study, the athlete gave a higher score to body satisfaction after mindful eating exercises than in the first interview and although she was a little dissatisfied with this issue, she stated that after the mindful raisin exercises, she had more positive thoughts about her body than before. Alberts et al. (2012) observed a decrease in the participants’ body image concerns after an 8-week MBCT (mindfulness-based cognitive therapy) study on 26 women with eating behavior disorders. This finding also indicates that mindful eating may serve as a buffer against the negative psychological impact of external pressures. Although there is no study showing that mindful eating behaviors directly reduce family pressure or social pressure on weight control.

The athlete responded to another theme, “perceived performance,” in the semi-structured interviews. She stated that mindful eating improved her performance, attributing this improvement to avoiding junk food. She also highlighted that unhealthy nutrition triggers the secretion of the fatigue hormone, suggesting that mindful eating helped her feel more energetic and enhanced her performance. In parallel with this study results, Augustus et al. (2024) worked with 55 student-athletes in their study examining the effects of mindfulness-based interventions (MBI) on the quality of life and performance of student-athletes. In the study, student-athletes reported an improvement in their perceived subjective performance in the interviews conducted after the 7-session mindfulness-based intervention.

In combat sports, when considering the psychological strategies used to help athletes cope with the pressures of weight control, third-wave psychotherapy approaches such as Mindfulness-Based Cognitive Therapy (MBCT) and Mindfulness-Based Stress Reduction (MBSR) have become more prominent in recent years compared to cognitive-behavioral methods (Omiwole et al., 2019).

Compared to cognitive-behavioral approaches, in the mindful eating method athletes do not struggle with or attempt to change their eating urges; instead, they approach these thoughts with distance and observe them (Alpay and Tuncel, 2022), This act of observation helps the athlete conserve mental energy as the approach does not require a constant effort to restructure thoughts.

However, as a limitation of this method, it is considered that the effects of mindful eating on eating behaviors may be more sustainable only if regular mindfulness exercises are maintained (Kristeller et al., 2014).

## Conclusion

In light of all this, mindful eating skills could be an effective approach to weight control, especially for the participant. This approach would also reduce the pressure on the athlete, who had to reach a certain weight to avoid disqualification.

However, more observations and research are needed to examine the mechanisms underlying difficulties in weight control in sports.

## Limitations

Since this study is a single-person case report, it has limitations in generalizing the results to other athletes and the adolescent population. Another limitation of this case is that the athlete had no prior experience with structured dietary programs under the supervision of a nutritionist. This may have influenced the athlete’s adherence to the dietary plan and could have had an impact on the outcomes observed in this intervention. Additionally, the individual characteristics, situations, and experiences of the athlete included in the study may be unique, which may make it difficult to disseminate the findings to a broad population. Because the findings are based on a single participant, the reliability of the data may be limited. With multiple participants, the reliability of the data can be increased by ensuring greater repeatability. Additionally, the study did not include a control group or repeated scale measurements, which could have strengthened the reliability and generalizability of the results. Future research could address these limitations by including multiple participants, control groups, and repeated assessments to better evaluate the effects of mindful eating interventions under competitive pressure.

## Author’s note

The abstract of the article was presented at the FEPSAC (European Federation of Sport Psychology) 17th Congress held in Innsbrück, Austria, between 15 and 19 July 2024.

## Data Availability

The original contributions presented in the study are included in the article/supplementary material, further inquiries can be directed to the corresponding author.
